# Diversity in knowledge search paths and breakthrough technological innovation: Evidence from new energy vehicle firms

**DOI:** 10.1371/journal.pone.0326736

**Published:** 2025-06-26

**Authors:** Xiao Yin, Le Song, Ying Pan

**Affiliations:** 1 School of Business Administration, South China University of Technology, Guangzhou, China; 2 School of Business, Shandong Normal University, Jinan, China; Fooyin University, TAIWAN

## Abstract

Breakthrough technological innovations have heralded a transformative era within the automotive industry. Knowledge search diversity, as the key to acquiring the raw materials for knowledge recombination, enables firms to overcome resource constraints and accelerate the realization of breakthrough technological innovations. This paper integrates the theories of organizational search and knowledge recombination theory and draws upon the relationship between knowledge and industry to distinguish between basic knowledge search diversity and applied knowledge search diversity. It further investigates the distinct relationships between these two forms of knowledge search diversity and their respective contributions to breakthrough technological innovations. We develop research hypotheses and employ a negative binomial fixed-effects regression model to empirically test data from 280 Chinese new energy vehicle (NEV) manufacturers. The results demonstrate that: (1) knowledge search diversity exhibits an inverted U-shaped relationship with breakthrough technological innovation; (2) the inverted U-shaped relationship is steeper for basic knowledge search diversity compared to applied knowledge search diversity; (3) organizational proximity positively moderates the inverted U-shaped relationship between knowledge search diversity and breakthrough technological innovation. The findings provide practical insights into optimizing knowledge search strategies for technological convergence and accelerating the internalization of external resources to foster innovative transformation and upgrading.

## 1 Introduction

Breakthrough technological innovations are fundamental drivers of new industries, business models, and sources of growth, playing a crucial role in enabling firms to secure sustainable competitive advantages. As the primary catalyst for innovation, knowledge forms the foundation of breakthrough technological advancements, with a diverse range of knowledge resources being essential to their realization [1]. For instance, in the new energy vehicle (NEV) industry, firms integrate knowledge from various domains, including energy, computer science, electronics, information technology, big data, and transportation. This convergence of disciplines fuels the continuous emergence of breakthrough innovations, triggering transformative shifts in the automotive sector. However, due to resource constraints, knowledge search emerges as a key mechanism for overcoming these limitations and facilitating breakthrough innovations [2,3]. Additionally, knowledge recombination theory suggests that the type and volume of available knowledge elements determine the opportunities and potential for effective knowledge recombination [4]. This highlights the growing necessity for firms to enhance the diversity of their knowledge search efforts. Investigating strategies to cultivate knowledge search diversity in order to strengthen breakthrough technological innovations is of significant practical importance, as it can enhance innovation quality and enable firms to achieve a competitive edge on the global stage.

Unlike incremental technological innovations, breakthrough innovations require firms to transcend established pathways and make disruptive changes to existing products or services, thus entering entirely new technological domains [5]. This necessitates a broad and comprehensive knowledge search [6]. Knowledge search theory suggests that diversity in knowledge search can enrich a firm’s knowledge base, with the acquisition of diverse and heterogeneous knowledge enabling firms to overcome technological bottlenecks. However, excessively diverse knowledge searches can impose significant costs and resource burdens, raising the question of how to effectively leverage knowledge search diversity to facilitate breakthrough technological innovations. To date, a consensus on this issue has yet to be reached. Moreover, scholars focused on R&D strategy have observed that exploring nascent, high-potential basic knowledge during the search process can reconstruct existing knowledge frameworks and is more likely to result in breakthrough inventions [7,8]. Unfortunately, current research has largely overlooked the impact of knowledge maturity on the realization of breakthrough innovations through technological integration. Based on the level of knowledge maturity, knowledge can be divided into basic and applied knowledge. Basic knowledge refers to systematic knowledge focused on theoretical research and exploration of principles in the field of science and technology. Applied knowledge refers to the transformation of basic knowledge into specific technologies and solutions in the process of practical application. In practice, because basic knowledge is often complex, obscure, and difficult to absorb [9,10], firms tend to focus on more mature, applied knowledge in an effort to mitigate risks and uncertainties. However, this strategy may simultaneously reduce the likelihood of achieving breakthrough technological innovations. In response, this paper highlights the relationship between knowledge and industry in the context of corporate knowledge search, distinguishing between basic and applied knowledge search diversity. It investigates how different knowledge search strategies influence both the quantity and quality of breakthrough technological innovation outcomes.

In the era of open innovation, relying solely on knowledge search diversity is insufficient for firms to independently develop breakthrough technologies in a timely manner. Collaborative R&D has emerged as an effective strategy for firms to rapidly acquire, understand, and apply cutting-edge knowledge, overcome technological challenges, and achieve technological integration [11]. However, technological innovation partnerships inherently increase the risks of losing proprietary knowledge, secrets, and control [12]. Proximity dynamics theory highlights the importance of organizational proximity, which refers to the relationships among members within the same organizational entity, such as subsidiaries or departments within the same parent company [13–15]. This proximity allows firms to strategically mitigate opportunism in collaborations by holding equity stakes in partner firms, thereby facilitating knowledge transfer and enhancing the opportunities for the recombination of heterogeneous knowledge [12]. However, from a resource-based perspective, firms often face constraints in terms of financial resources and limited channels for sourcing knowledge, which hampers their ability to access diverse new knowledge and restricts their capacity to recombine knowledge for breakthrough technological innovation. As firms strive for breakthrough technological innovations, they must determine whether to rely on highly proximate organizational partners to solve emerging challenges in unfamiliar domains or to engage a broader set of external actors to accelerate problem-solving. This remains an unresolved issue. Consequently, this study examines the moderating role of organizational proximity in the relationship between knowledge search diversity and breakthrough technological innovation, providing deeper insights into how proximity influences innovation outcomes.

In summary, this paper draws on the theory of knowledge search and knowledge recombination to analyse the relationship between knowledge search diversity and breakthrough technological innovation and the moderating role of organizational proximity using a negative binomial regression fixed effects model for Chinese new energy vehicle (NEV) manufacturing firms from 2010-2019. *Breakthrough technological innovation (BTI)* refers to innovation activities through which firms achieve a substantial qualitative leap in their technological trajectories [1,5]. Knowledge search diversity can be categorized into *basic knowledge search diversity (BASIC_KSD)* and *applied knowledge search diversity (APPLIED_KSD)*. The former refers to the degree of heterogeneity in seeking theoretical and fundamental knowledge across different scientific domains, and is typically measured by the diversity of academic paper citations [16,17]. The latter refers to the degree of heterogeneity in acquiring specific applied knowledge across various technological domains, commonly represented by the classification of patents [18,19]. *Organizational proximity (OP)* refers to affiliation-based relationships between organizations, such as subsidiaries or departments under the same parent company [13–15]. To further clarify these concepts, we present a summary in Table 1. Our study adopts a negative binomial fixed-effects regression model, which is particularly well-suited for the analysis of count data exhibiting overdispersion. This methodological approach enhances the robustness and reliability of our findings, addressing limitations associated with traditional linear models frequently employed in prior research. Furthermore, our utilization of panel data spanning a substantial period (2010-2019) enables us to capture the dynamic evolution of innovation processes. This long-term perspective offers a more nuanced and comprehensive understanding of the relationship between knowledge search diversity and breakthrough technological innovation. Compared to studies that rely on shorter timeframes or smaller sample sizes, our data selection significantly enhances the reliability and generalizability of our findings.”

**Table 1:** Main variables and definitions.

Main variables	Definitions
*BTI*	Innovation activities through which firms achieve a substantial qualitative leap in their technological trajectories [1,5].
*BASIC_KSD*	The degree of heterogeneity in seeking theoretical and fundamental knowledge across different scientific domains, and is typically measured by the diversity of academic paper citations [16,17].
*APPLIED_KSD*	The degree of heterogeneity in acquiring specific applied knowledge across various technological domains, commonly represented by the classification of patents [18,19].
*OP*	Affiliation-based relationships between organizations, such as subsidiaries or departments under the same parent company [13–15].

Specifically, the study addresses two key research questions: (1) How do different knowledge search diversity strategies influence breakthrough technological innovation during the process of technological integration? (2) What role does organizational proximity play in determining the extent to which diverse knowledge search strategies enhance firms’ breakthrough technological innovation? Our study makes several significant theoretical contributions to the field. First, we differentiate between basic and applied knowledge search diversity, offering a more granular perspective on how distinct types of knowledge search influence innovation. Second, we examine the moderating effect of organizational proximity on the relationship between knowledge search diversity and breakthrough technological innovation, underscoring the critical role of contextual factors in shaping innovation outcomes. These findings advance existing theories on knowledge search and breakthrough technological innovation by integrating the influence of organizational proximity and the dynamics of technological convergence.

## 2 Literature review and research hypotheses

Breakthrough technological innovation refers to innovation activities through which firms achieve a significant qualitative shift in their technological trajectory. In contrast to incremental innovation, breakthrough innovation places a stronger emphasis on the exploration of new knowledge [5]. Knowledge resources are vital strategic assets for firms, and knowledge search diversity indicates the extent to which a firm acquires heterogeneous knowledge from multiple domains [2,8]. By enriching the firm’s knowledge base, this diversity increases the likelihood of knowledge recombination, thereby impacting the performance of breakthrough technological innovation [20]. In the context of the relationship between knowledge and industry, knowledge can be categorized into basic and applied knowledge [16,21]. Basic knowledge refers to concepts, principles, theories, and facts that reveal the underlying logic of phenomena and observable facts without any immediate practical application. Typically disseminated through academic papers, basic knowledge is foundational and often lacks direct utility. In contrast, applied knowledge involves the use of basic knowledge to address practical problems and operational challenges. It is typically associated with specific applications, yielding tangible economic benefits, and is often protected by patents [21]. Overall, basic knowledge serves as the cornerstone of the knowledge system, while applied knowledge is the process of transforming these foundational insights into practical capabilities. The two are mutually complementary and equally essential. By strategically deploying different knowledge search diversity strategies, firms can reorganize their knowledge systems, thereby facilitating the realization of breakthrough technological innovations.

### 2.1 Basic knowledge search diversity and breakthrough technological innovation

In line with the nonlinear dynamics of scientific development and the evolution of technological trajectories, breakthrough technological innovation is characterized by a sudden shift in the intensity and extent of technological change, driven by breakthroughs in scientific knowledge or principles [22]. Such innovations play a pivotal role in overcoming technological bottlenecks. Basic knowledge search diversity enables firms to acquire advancements from multiple scientific disciplines, allowing them to reconstruct foundational technological [3,7,22]. This, in turn, creates opportunities for knowledge recombination during the early stages of technological development, adjusting the interdependencies within existing technology systems and facilitating breakthrough innovations.

However, as with the “two sides of a coin”, basic knowledge search presents a challenge due to the distance between fundamental knowledge and its eventual technological application. For firms, excessive searches and the conversion of technological knowledge into actionable innovation incur significant time and economic costs. On one hand, many firms lack the financial resources to absorb the considerable costs associated with overly broad knowledge searches [17]. On the other hand, in a fast-paced, highly competitive market, extended research cycles can lead to missed opportunities, hindering the achievement of breakthrough technological outcomes [10].

A case in point is Tesla, which, after successfully developing its battery pack, became fixated on introducing a secondary gearbox. However, the relevant technology was not yet mature, requiring extensive searches for frontier theoretical knowledge and the restructuring of basic knowledge systems to launch systematic R&D. This resulted in escalating costs and prolonged development cycles, nearly driving Tesla to the brink of failure. Subsequently, Tesla’s strategic narrowing and targeting of its search scope resulted in groundbreaking innovations. This example underscores the “double-edged sword” nature of basic knowledge search diversity. On one hand, a high level of basic knowledge search diversity ensures that firms develop a more robust theoretical knowledge base, enhancing the probability of knowledge reorganization and providing new solutions to technical challenges, thereby facilitating breakthrough technological innovations. On the other hand, identifying and acquiring frontier basic knowledge involves substantial costs. When knowledge search diversity reaches a certain threshold, transforming basic knowledge into mature technology often requires extensive trial and error. Firms may struggle to absorb and apply this knowledge, leading to inefficiencies and wasted resources, which ultimately hinder the realization of breakthrough technological innovations [10,17]. Therefore, we propose the following hypothesis:

**H1a: Basic knowledge search diversity has an inverted U-shaped relationship with the quantity of firms’ breakthrough technological innovation.**

Basic research is a fundamental source of breakthrough innovations in industry, serving as the primary avenue through which firms explore untapped technological domains [3]. Basic knowledge search diversity enables firms to access cutting-edge scientific principles across a range of disciplines, allowing them to develop a more comprehensive understanding of the technological landscape. This, in turn, guides firms toward more promising technological pathways, fostering the creation of impactful and breakthrough innovations [7,18].

However, the transition from basic knowledge to technological maturity is typically a lengthy trial-and-error process. The encoding of basic knowledge often differs substantially from a firm’s internal knowledge structures, making it challenging to assimilate and apply. Basic knowledge is often abstract and complex, and excessive diversity in its acquisition can intensify the difficulties associated with knowledge conversion [10]. Moreover, the novelty inherent in integrating diverse basic knowledge can heighten the tension between knowledge discovery and technological development [23], making it difficult for firms to generate high-quality breakthrough innovations.

A striking example of this contrast is the outcome of two major communication projects: the Iridium project and the Starlink project. Both aimed to revolutionize satellite communication, yet their results were drastically different. The Iridium project was launched prematurely when relevant technologies were still in their nascent stages. This required an extensive exploration of basic knowledge across multiple domains, coupled with limited understanding of the commercial potential, leading to its eventual failure. In contrast, the Starlink project was built on a foundation of more mature technologies, significantly narrowing the scope of its basic knowledge search. By leveraging more data to refine its business model, Starlink was able to close the gap between basic knowledge and industrial application, leading to high-quality breakthrough innovations, such as low-cost satellite launches.

In conclusion, this paper argues that there is an inverted U-shaped relationship between basic knowledge search diversity and the quality of innovation in firms. On the one hand, basic knowledge search diversity helps build a robust knowledge system, enabling firms to anticipate future trends and identify promising technological pathways with high developmental potential. On the other hand, excessive search diversity incurs significant costs. Beyond the increased expenditure required to acquire excessive basic knowledge, firms must also invest considerable resources in trial and error, which amplifies the uncertainty and risks associated with technological development. As a result, this elevated complexity hampers the firm’s ability to generate high-quality breakthrough technological innovation outcomes. Thus, we propose the following hypothesis:

**H1b: Basic knowledge search diversity has an inverted U-shaped relationship with the quality of firms’ breakthrough technological innovation.**

### 2.2 Applied knowledge search diversity and breakthrough technological innovation

Corporate innovation activities necessitate a foundational understanding of industry-specific knowledge, including the acquisition of external knowledge from other firms within the industry, which provides direction, methodological guidance, and evaluation metrics for technological innovation. Applied knowledge search provides firms with industry-specific technical knowledge, including improvements in production skills, process innovation, and workflow optimization, ensuring that new concepts are translated into innovative products [8]. Unlike industries primarily based on scientific knowledge, industries characterized by complex technological systems—such as the NEV sector—rely predominantly on synthetic knowledge [14]. Diverse applied knowledge serves as the basis for turning concepts into products. Since basic knowledge lacks a specific commercialization purpose, it is essential to combine basic research with applied research to ensure that basic knowledge aligns with market needs and provides innovative, effective, and safe new solutions [21]. Applied knowledge search diversity helps firms monitor technological trends, supports R&D strategies [19], and offers more opportunities for knowledge recombination [18]. Through the secondary development of technologies, firms can refine existing technologies and extend their capabilities [19]. Moreover, the relatively mature characteristics of applied knowledge help reduce innovation risks and uncertainties [24], enhancing firms’ efficiency in restructuring, absorbing, and utilizing diverse applied knowledge, thereby increasing the likelihood of high-quality innovation outputs. However, conducting such searches requires significant costs, and excessive applied knowledge search diversity can become a burden for firms [17]. Additionally, applied knowledge often serves a specific purpose [21], focuses on practical problems, and has established technological barriers. When applied knowledge search diversity is too high, the difficulty of integrating applied knowledge increases significantly, potentially resulting in an inability to escape existing R&D trajectories or challenges in focusing effectively. Such difficulties can ultimately hinder technological innovation. Therefore, we propose the following hypothesis:

**H2a: Applied knowledge search diversity has an inverted U-shaped relationship with the quantity of firms’ breakthrough technological innovation.**

Extensive applied knowledge search diversity can bring heterogeneous new knowledge, stimulating employees’ innovative thinking. Distant recombination is an important pathway for firms to explore unknown technological fields [3]. Rich applied knowledge search diversity can effectively help firms overcome capability traps, challenge existing cognitive inertia, and significantly enhance breakthrough innovation [25]. Additionally, applied knowledge search diversity assists firms in tracking new technological trends in the market, avoiding redundant investments in existing technologies, and enhancing the novelty of innovation outputs [19]. However, excessive pursuit of applied knowledge search diversity may dilute managerial attention on critical knowledge while incurring high search costs and redundant resources [26]. Wide-ranging applied knowledge searches require substantial costs for identifying, integrating, absorbing, and utilizing these diverse knowledge resources. Given its specific applicability, applied knowledge may not sufficiently diffuse throughout the organization without in-depth understanding, ultimately reducing innovation efficiency and quality. Furthermore, excessive applied knowledge search increases the difficulty of integrating knowledge resources, leading to effective knowledge being buried, with unintegrated knowledge becoming redundant, ultimately hindering the production of high-quality innovations. Therefore, we propose the following hypothesis:

**H2b: Applied knowledge search diversity has an inverted U-shaped relationship with the quality of firms’ breakthrough technological innovation.**

For firms, conducting both basic and applied knowledge searches is essential for building internal scientific capabilities. However, firms must bear substantial costs, risks, and time commitments [16]. Compared to applied knowledge search diversity, basic knowledge search diversity provides firms with fundamental principles, offering more novel and broadly applicable solutions. Nevertheless, basic knowledge’s lack of specific application purposes leads to longer transformation cycles and heightened uncertainty and opportunism [22]. Conversely, applied knowledge features relatively higher technological maturity [24], enabling heterogeneous applied knowledge to be more readily converted into innovations. However, compared to basic knowledge, applied knowledge is more limited in scope, reducing opportunities for knowledge recombination, with recombination thinking constrained by established paths, thus making breakthrough innovations more difficult to achieve. Therefore, although basic knowledge search diversity involves greater challenges in transformation, when breakthroughs do occur, they lead to significant advancements in breakthrough technological innovation. Based on this, we propose the following hypotheses:

**H2c: Compared to applied knowledge search diversity, basic knowledge search diversity has a steeper inverted U-shaped relationship with the quantity of firms’ breakthrough technological innovation.**

**H2d: Compared to applied knowledge search diversity, basic knowledge search diversity has a steeper inverted U-shaped relationship with the quality of firms’ breakthrough technological innovation.**

### 2.3 The moderating role of organizational proximity

The sources of knowledge for breakthrough technologies extend beyond the boundaries of the organization; the diversity of knowledge necessary for their recombination may also stem from external organizations. Balland *et al*. [13] define organizational proximity as the relationships between organizational entities, such as subsidiaries or departments under the same parent company. For example, HiSilicon, a first-tier core subsidiary of Huawei Group, focuses on cutting-edge research and innovation in chip design, while the Central Software Institute, a second-tier unit within Huawei’s R&D division, is primarily responsible for terminal software development. Both departments are under the unified management of Huawei Group, and this tightly integrated organizational structure allows them to fully leverage the advantages of organizational proximity. Each week, the two units hold regular cross-departmental technical meetings via internal collaboration platforms to exchange updates and align technological needs. This seamless coordination significantly shortens the adaptation cycle between 5G chips and mobile operating systems—far outperforming traditional collaboration models with external partners—while maintaining full control over core technologies.

The proximity dynamics perspective highlights that shared affiliation within a parent company fosters a sense of identity between organizations [27], facilitating knowledge transfer. This proximity enables the coordination of innovation activities, reduces trial-and-error, and accelerates the delivery of breakthrough innovations [15,28]. For example, Google operates multiple innovation labs, such as Google X, which played a crucial role in the development of autonomous driving technology. During this process, Google X worked closely with various departments, including software development, data analytics, and hardware manufacturing. These departments conducted extensive basic and applied knowledge searches, spanning areas like artificial intelligence algorithms, big data processing, sensor technologies, and automotive engineering. Organizational proximity allowed for rapid knowledge and resource sharing across departments, which, in turn, facilitated breakthrough innovations in autonomous driving. Thus, a high degree of organizational proximity aligns the interests of collaborating entities, preventing opportunistic behavior, safeguarding proprietary information, fostering knowledge exchange, reducing transaction costs, and ultimately enhancing the efficiency of breakthrough technological innovations. Based on the above discussion, we propose the following hypotheses:

**H3: Organizational proximity positively moderates the inverted U-shaped relationship between knowledge search diversity and the quantity of firms’ breakthrough technological innovation.**

**H3a: Organizational proximity positively moderates the inverted U-shaped relationship between basic knowledge search diversity and the quantity of firms’ breakthrough technological innovation.**

**H3b: Organizational proximity positively moderates the inverted U-shaped relationship between applied knowledge search diversity and the quantity of firms’ breakthrough technological innovation.**

Breakthrough technological innovation involves disrupting existing cognitive frameworks through exploratory learning and innovation [5]. And exploratory learning should consider more comprehensively the role of technological partners in facilitating technology exploration [27]. Actors with high organizational proximity belong to the same relational network, where similar organizational cultures and practices create an environment conducive to information exchange [9]. Trust among members enhances interactions between innovation actors, facilitating the acquisition, dissemination, absorption, and integration of diverse and heterogeneous knowledge. This, in turn, amplifies the effect of knowledge search diversity on breakthrough innovation, catalyzing high-quality outcomes [29]. Moreover, strong affiliations ensure that organizations can coordinate innovation initiatives [12]. While acquiring diverse knowledge, they also focus on shared, frontier challenges, thereby reducing the coordination costs associated with R&D communication [28]. This allows R&D personnel to concentrate on critical technological advancements, significantly improving the efficiency of knowledge recombination and transformation. For example, China National Nuclear Corporation (CNNC) leveraged its group resources to create the “Source-Device” collaborative model, acquiring diverse basic and applied knowledge and enabling the deep integration of nuclear technology and medical equipment. This led to the successful independent development of the domestically produced new gamma-ray stereotactic radiotherapy system, marking a significant breakthrough in China’s high-end medical equipment sector. Based on these insights, we propose the following hypothesis:

**H4: Organizational proximity positively moderates the inverted U-shaped relationship between knowledge search diversity and the quality of firms’ breakthrough technological innovation.**

**H4a: Organizational proximity positively moderates the inverted U-shaped relationship between basic knowledge search diversity and the quality of firms’ breakthrough technological innovation.**

**H4b: Organizational proximity positively moderates the inverted U-shaped relationship between applied knowledge search diversity and the quality of firms’ breakthrough technological innovation.**

**Fig 1:**
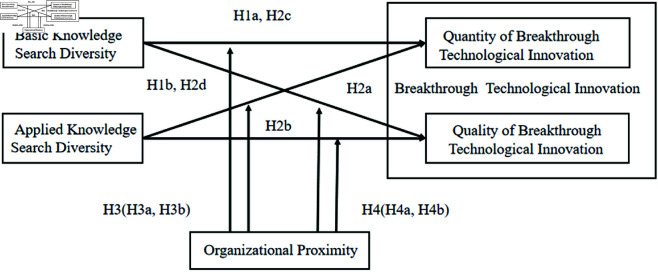
Research model.

In summary, this study proposes a theoretical model that links knowledge search diversity, organizational proximity, and breakthrough technological innovation, as depicted in Fig 1.

## 3 Research design

### 3.1 Sample selection and data sources

This study considers two primary factors when selecting the data. First, breakthrough technological innovation has enabled China’s automotive industry to overcome foreign technological barriers and create new competitive opportunities, with the development of new energy vehicles (NEVs) being a strategic choice for the high-quality growth of the sector. Moreover, NEVs encompass a wide range of engineering and technological knowledge, highlighting the need for companies to adopt effective knowledge search strategies to facilitate knowledge conversion and achieve breakthrough innovations. Second, China has become the world’s largest NEV market. According to the China Association of Automobile Manufacturers, by 2023, the market share of domestically produced NEVs had reached 31.6%, continuing its rapid growth and maintaining the top global position for nine consecutive years. China’s NEV industry is asserting itself as a global leader in the sector [30]. Thus, The study of the impact of knowledge search diversity on breakthrough technological innovations in Chinese companies in the new energy vehicle industry is typical and provides access to abundant and reliable publicly available data. This study utilizes data from the Chinese NEV industry, sourced from the “Announcement of Vehicle Manufacturing Enterprises and Products” issued by China’s Ministry of Industry and Information Technology. We collected data from the first to the 355th batch of enterprises spanning from 2008 to 2022, initially identifying 354 NEV manufacturers based on fuel type. Additionally, patent data and operational business data—including firm age and ownership structure—were obtained from the PatSnap global patent database, TianYanCha, and the National Enterprise Credit Information Publicity System. Finally, companies with substantial missing data or those that acquired patents primarily through rights transfer were excluded, resulting in a final sample of 280 NEV manufacturers.

### 3.2 Variable measurement

#### 3.2.1 Independent variables.

**(1) *Basic knowledge search diversity (BASIC_KSD).*** A common measure for interdisciplinary domains is the number of disciplines involved [18]. In this study, experts in the NEV sector were invited to identify and filter the invention patent data of the sample firms, excluding non-breakthrough patents. Basic knowledge search diversity was measured using the Herfindahl index, based on the Chinese Library Classification (CLC) of the cited literature following breakthrough invention patents. The calculation is given by $1-\sum {\left(\frac{N_{i}}{N}\right)}^{2}$, where *N*_*i*_ represents the number of times patents in discipline *i* are cited in a given year, and *N* is the total number of breakthrough invention patent applications for that year.

**(2) *Applied knowledge search diversity (APPLIED_KSD).*** Applied knowledge search diversity was measured using the four-digit IPC codes of patents citing the firm’s breakthrough patents during a given year, employing the Herfindahl index [17,19]. The calculation is given by $1-\sum {\left(\frac{M_{i}}{N}\right)}^{2}$, where *M*_*i*_ represents the number of times patents in technology field *i* are cited in a given year, and *N* is the total number of breakthrough invention patent applications for that year.

#### 3.2.2 Dependent variables.

***Breakthrough technological innovation (BTI).*** In line with prior research, *the quantity of breakthrough technological innovation (QUAN_BTI)* was measured by the annual number of breakthrough invention patent applications identified by NEV technology experts. *The quality of breakthrough technological innovation (QUAL_BTI)* was assessed based on the cumulative number of citations of these breakthrough patents over the preceding five years.

#### 3.2.3 Moderating variable.

***Organizational proximity (OP).*** When entities share affiliations or exhibit structural similarities, they are more likely to engage in interactive behaviors. This study adopts a narrow definition of organizational proximity, specifically referring to affiliations between entities. Organizational proximity was measured by setting a dummy variable based on the capital linkages between partner organizations. Data on organizational proximity were gathered from multiple sources, including the National Enterprise Credit Information Publicity System, company websites, and Tianyancha, and were classified into six levels. The proximity levels were assigned sequential values from 1 to 6, where a value of 6 represents wholly-owned subsidiaries, 5 represents majority-owned subsidiaries, 4 represents minority-owned subsidiaries, 3 represents firms owned by the same investment company, 2 represents partner firms that are parent companies, and 1 represents general external collaborations [15].

#### 3.2.4 Control variables.

Additional variables were included to improve the reliability of our analysis. Firstly, we controlled for firm age to account for the potential impact of organizational flexibility on innovation [31]. Secondly, we included the firm’s patent stock to control for the influence of technological accumulation on innovation outcomes [32]. Thirdly, acknowledging that a larger number of partners can expand a firm’s knowledge base while also increasing management costs, we considered cooperation scale as a control variable [25]. Fourthly, as companies often acquire new knowledge and human capital through acquisitions to improve their ability to generate valuable new knowledge, we included a dummy variable to control for whether the firm engaged in an acquisition during the year [33,34]. Finally, we controlled for the scale of both basic and applied knowledge searches to account for the effects of knowledge base expansion on innovation [2,8]. On the whole, we introduced six control variables: *firm age (FA), patent stock (PS), cooperation scale during the year (CS_YEAR), merger and acquisition activities during the year (MAA_YEAR), basic knowledge search scale (BASIC_KSS), and applied knowledge search scale (APPLIED_KSS)*. Detailed information regarding the control variables used in the study is provided in Table 2.

**Table 2:** Control variables and measures.

Control variables	Measures
*FA*	Measured by the number of years from the firm’s establishment to 2022.
*PS*	The total number of breakthrough invention patents before 2010.
*CS_YEAR*	The total number of entities owning the patent rights for breakthrough invention patents in a given year.
*MAA_YEAR*	A dummy variable indicating whether the firm engaged in mergers or acquisitions in a given year (1 if yes, 0 if no).
*BASIC_KSS*	Measured as the ratio of the number of academic paper citations to the number of breakthrough patent applications in a given year. A higher value indicates greater absorption of scientific knowledge by the firm.
*APPLIED_KSS*	Measured as the ratio of the number of patent citations to the number of breakthrough patent applications in a given year. A higher value indicates greater absorption of technological knowledge by the firm.

### 3.3 Model selection

In this study, “breakthrough technological innovation” is treated as the dependent variable. Since it is a non-negative count variable, ordinary least squares (OLS) regression is not suitable. Instead, Poisson regression or negative binomial regression is more appropriate [35]. Moreover, as the variance of the dependent variable is significantly larger than its mean (as shown in Table 3), overdispersion is present, which violates the assumption of Poisson regression that the mean should equal the variance. As a result, we opted for a negative binomial regression model. Additionally, a Hausman test was conducted [36], following the methodology of scholars such as Lian *et al*. [37], and based on the results, we selected a fixed-effects panel negative binomial regression model for the analysis.

**Table 3:** Descriptive statistics and correlation coefficients of variables.

Variables	Mean	SD	VIF	1	2	3	4	5	6	7	8	9	10
*QUAN_BTI*	13.133	52.293		1.000									
*QUAL_BTI*	42.889	215.702		0.860^***^	1.000								
*BASIC_KSD*	0.058	0.175	1.71	0.590^***^	0.531^***^	1.000							
*APPLIED_KSD*	0.314	0.394	2.26	0.375^***^	0.302^***^	0.492*^***^	1.000						
*OP*	0.252	0.918	1.48	0.160^***^	0.135^***^	0.282^***^	0.311^***^	1.000					
*BASIC_KSS*	0.064	0.345	1.24	0.046^**^	0.046^**^	0.304^***^	0.178^***^	0.072^***^	1.000				
*APPLIED_KSS*	2.385	3.822	1.95	0.183^***^	0.150^***^	0.290^***^	0.652^***^	0.195^***^	0.337^***^	1.000			
*CS_YEAR*	0.205	0.880	1.56	0.329^***^	0.230^***^	0.339^***^	0.295^***^	0.541^***^	0.059^**^	0.159^***^	1.000		
*FA*	19.695	9.904	1.04	0.080^***^	0.070^***^	0.102^***^	0.119^***^	0.127^***^	0.009	0.061^**^	0.161^***^	1.000	
*PS*	52.175	279.879	1.25	0.737^***^	0.615^***^	0.395^***^	0.227^***^	0.141^***^	0.033^*^	0.117^***^	0.269^***^	0.100^***^	1.000
*MAA_YEAR*	0.136	0.343	1.08	0.234^***^	0.232^***^	0.208^***^	0.151^***^	0.120^***^	0.010	0.063^***^	0.078^***^	0.046^**^	0.204^***^

**Note:** ^*^p < 0.1, ^**^p < 0.05, ^***^p < 0.01.

## 4 Empirical results analysis

### 4.1 Descriptive statistics and correlation analysis

The empirical analysis was conducted using Stata software, and all results presented herein were generated using Stata. Descriptive statistics and correlation analysis were conducted for all variables involved in the study. The means, standard deviations, variance inflation factor (VIF) values, and Pearson correlation coefficients for all variables are presented in Table 3. The results indicate that the correlation coefficients between variables are generally below 0.8, while the correlation coefficients between independent variables are below 0.6. The maximum VIF value is 2.26, which is well below the threshold of 5, suggesting that multicollinearity is not a concern in this study. Therefore, the regression results are statistically reliable.

### 4.2 Regression results

#### 4.2.1 Impact on the quantity of breakthrough technological innovation.

Fixed-effects panel negative binomial regression was conducted, with basic knowledge search diversity and applied knowledge search diversity as independent variables, the quantity of breakthrough technological innovation as the dependent variable, and organizational proximity as a moderating variable. The regression results are presented in Table 4. Model 1 includes only the control variables; Models 2 and 3 include both the control variables and independent variables; Models 4 and 5 include the control variables, independent variables, the moderating variable, and interaction terms.

**Table 4:** Fixed-effects panel regression results (the quantity of breakthrough technological innovation).

Variables	Model 1	Model 2	Model 3	Model 4	Model 5
*FA*	0.0175^***^	0.0200^***^	0.0106^**^	0.0201^***^	0.0109^**^
*PS*	0.0001^*^	0.0001^*^	0.0002^***^	0.0001^**^	0.0002^***^
*CS_YEAR*	0.1863^***^	0.1027^***^	0.0793^***^	0.0952^***^	0.0761^***^
*MAA_YEAR*	0.3756^***^	0.2655^***^	0.1561^***^	0.2256^***^	0.1422^***^
*BASIC_KSS*	–1.0474^***^	–1.5122^***^	–0.1296^*^	–1.3475^***^	–0.1325^*^
*APPLIED_KSS*	0.0888^***^	0.0993^***^	–0.0141^*^	0.0883^***^	–0.0177^**^
*BASIC_KSD*		5.2667^***^		5.9564^***^	
*BASIC_KSD^2^*		–3.8632^***^		–4.5008^***^	
*APPLIED_KSD*			3.5246^***^		3.7013^***^
*APPLIED_KSD^2^*			–1.7937^***^		–1.8931^***^
*OP*				0.3796^***^	0.7580^***^
*OP × BASIC_KSD*				–1.4579^***^	
*OP × BASIC_KSD^2^*				1.3497^**^	
*OP × APPLIED_KSD*					–1.9449^***^
*OP × APPLIED_KSD^2^*					1.2194^***^
Constant	–1.6664^***^	–1.7853^***^	–2.4995^***^	–1.8179^***^	–2.5429^***^
Log likelihood	–4707.2496	–4529.1612	–3802.2349	–4464.0993	–3770.8298
Wald Chi-square	736.85	1245.92	1917.07	1376.90	1929.70
N	280	280	280	280	280

**Note:** ^*^p < 0.1, ^**^p < 0.05, ^***^p < 0.01.

Model 1 reveals that the control variables have a significant positive impact on the quantity of breakthrough technological innovation. Model 2 suggests that basic knowledge search diversity exhibits an inverted U-shaped relationship with the quantity of breakthrough technological innovation (linear term positive, quadratic term negative, p < 0.01), supporting hypothesis H1a. Similarly, Model 3 shows that applied knowledge search diversity also demonstrates an inverted U-shaped relationship with the quantity of breakthrough technological innovation (linear term positive, quadratic term negative, p < 0.01), supporting hypothesis H2a. The absolute value of the quadratic term in Model 2 is significantly greater than that in Model 3, and the slope derived from Model 2 is steeper than that of Model 3, indicating that basic knowledge search diversity has a more pronounced inverted U-shaped relationship with the quantity of breakthrough technological innovation compared to applied knowledge search diversity, thereby supporting hypothesis H2c.

**Fig 2:**
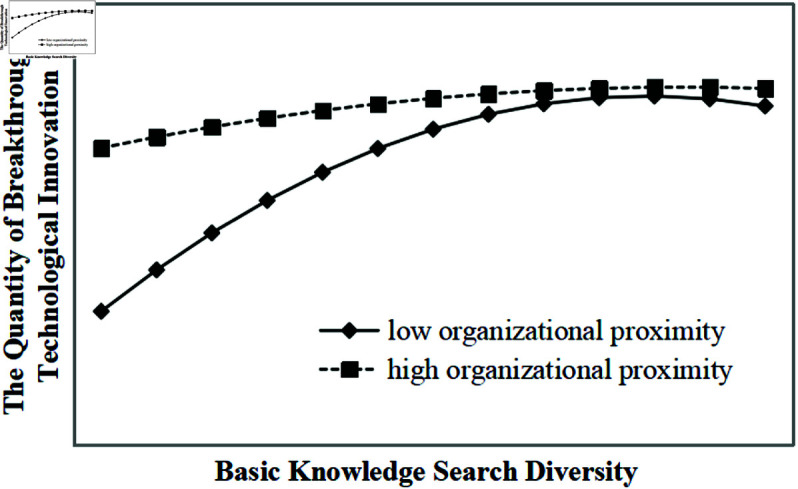
Moderating effect of organizational proximity on the inverted U-shaped relationship between basic knowledge search diversity and the quantity of breakthrough technological innovation.

**Fig 3:**
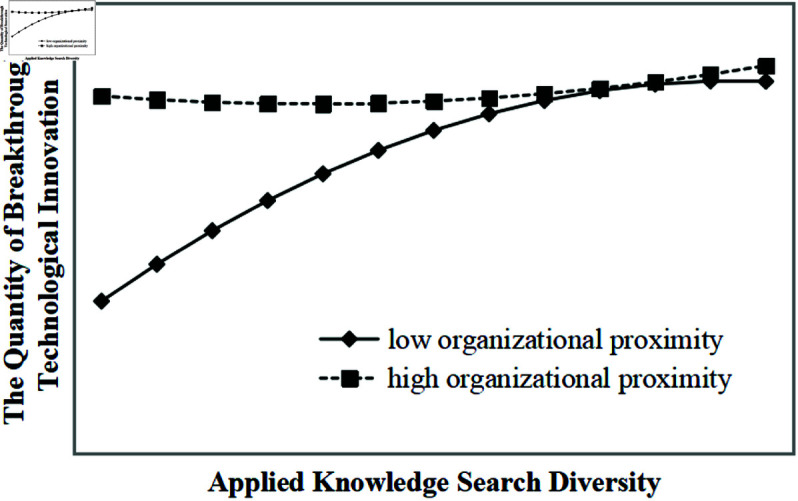
Moderating effect of organizational proximity on the inverted U-shaped relationship between applied knowledge search diversity and the quantity of breakthrough technological innovation.

Model 4 demonstrates that organizational proximity and its interaction terms with basic knowledge search diversity significantly moderate the inverted U-shaped relationship between basic knowledge search diversity and the quantity of breakthrough technological innovation (all p-values < 0.01). As illustrated in Fig 2, under the same level of basic knowledge search diversity, higher organizational proximity corresponds to a greater quantity of breakthrough technological innovation, supporting hypothesis H3a. Similarly, Model 5 shows that organizational proximity significantly moderates the inverted U-shaped relationship between applied knowledge search diversity and the quantity of breakthrough technological innovation (all p-values < 0.01). As illustrated in Fig 3, under the same level of applied knowledge search diversity, higher organizational proximity corresponds to a greater quantity of breakthrough technological innovation, supporting hypotheses H3b and H3.

#### 4.2.2 Impact on the quality of breakthrough technological innovation.

Fixed-effects panel negative binomial regression was also conducted, with basic knowledge search diversity and applied knowledge search diversity as independent variables, the quality of breakthrough technological innovation as the dependent variable, and organizational proximity as a moderating variable. The regression results are presented in Table 5. Model 6 includes only the control variables; Models 7 and 8 include both the control variables and independent variables; Models 9 and 10 include the control variables, independent variables, the moderating variable, and interaction terms.

**Table 5:** Fixed-effects panel regression results (the quality of breakthrough technological innovation).

Variables	Model 6	Model 7	Model 8	Model 9	Model 10
*FA*	0.0178^***^	0.0190^***^	0.0084^*^	0.0193^***^	0.0088^*^
*PS*	0.0009	–0.0001	–0.0001	–0.0001	–0.0001
*CS_YEAR*	0.1687^***^	0.0807^***^	0.0448^***^	0.0608^***^	0.0408^**^
*MAA_YEAR*	0.4062^***^	0.2293^***^	0.1542^**^	0.1973^***^	0.1345^**^
*BASIC_KSS*	–1.0971^***^	–1.5806^***^	0.0301	–1.3979^***^	0.0376
*APPLIED_KSS*	0.0951^***^	0.1043^***^	–0.0052	0.0925^***^	–0.0066
*BASIC_KSD*		6.3766^***^		6.7247^***^	
*BASIC_KSD^2^*		–4.8951^***^		–4.9184^***^	
*APPLIED_KSD*			5.5527^***^		5.6838^***^
*APPLIED_KSD^2^*			–3.5269^***^		–3.6207^***^
*OP*				0.4152^***^	0.8483^***^
*OP × BASIC_KSD*				–1.3084^***^	
*OP × BASIC_KSD^2^*				1.0026^***^	
*OP ×APPLIED_KSD*					–2.3917^***^
*OP × APPLIED_KSD^2^*					1.6056^***^
Constant	–2.2696^***^	–2.3857^***^	–3.3423^***^	–2.4365^***^	–3.3979^***^
Log likelihood	–5459.7876	–5261.9947	–4485.1689	–5200.9386	–4459.6403
Wald Chi-square	784.49	1327.38	1747.19	1419.27	1743.07
N	280	280	280	280	280

**Note:** ^*^p < 0.1, ^**^p < 0.05, ^***^p < 0.01.

Model 7 indicates that basic knowledge search diversity exhibits an inverted U-shaped relationship with the quality of breakthrough technological innovation (linear term positive, quadratic term negative, p < 0.01), supporting hypothesis H1b. Similarly, Model 8 shows that applied knowledge search diversity also demonstrates an inverted U-shaped relationship with the quality of breakthrough technological innovation (linear term positive, quadratic term negative, p < 0.01), supporting hypothesis H2b. The absolute value of the quadratic term in Model 7 is significantly greater than that in Model 8, and the slope derived from Model 7 is steeper than that of Model 8, indicating that basic knowledge search diversity has a more pronounced inverted U-shaped relationship with the quality of breakthrough technological innovation compared to applied knowledge search diversity, thereby supporting hypothesis H2d.

**Fig 4:**
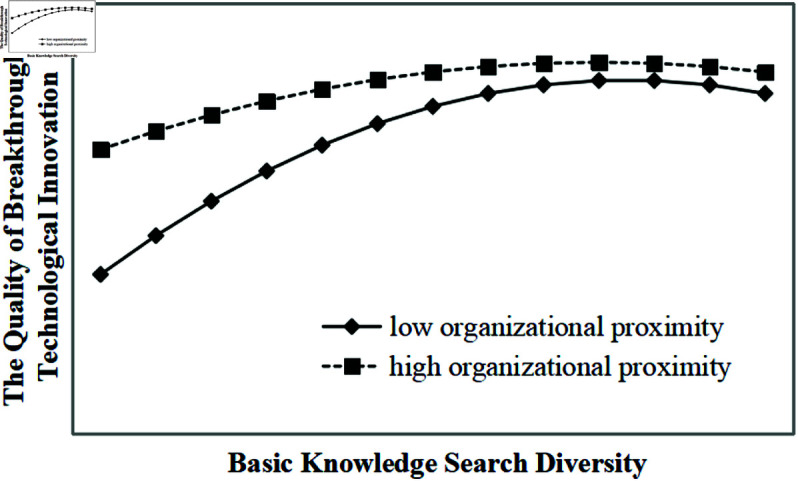
Moderating effect of organizational proximity on the inverted U-shaped relationship between basic knowledge search diversity and the quality of breakthrough technological innovation.

**Fig 5:**
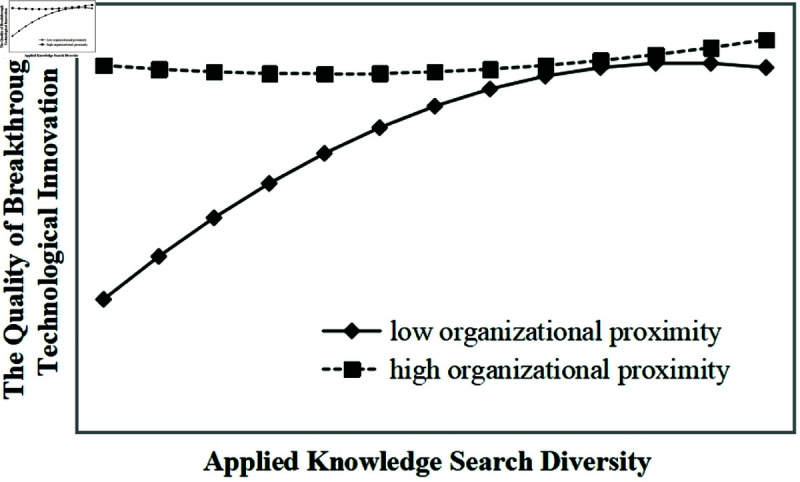
Moderating effect of organizational proximity on the inverted U-shaped relationship between applied knowledge search diversity and the quality of breakthrough technological innovation.

Model 9 shows that organizational proximity and its interaction terms with basic knowledge search diversity significantly moderate the inverted U-shaped relationship between basic knowledge search diversity and the quality of breakthrough technological innovation (all p-values < 0.01). As illustrated in Fig 4, under the same level of basic knowledge search diversity, higher organizational proximity corresponds to a higher level of breakthrough innovation quality, supporting hypothesis H4a. Similarly, Model 10 shows that organizational proximity significantly moderates the inverted U-shaped relationship between applied knowledge search diversity and the quality of breakthrough technological innovation (all p-values < 0.01). As illustrated in Fig 5, under the same level of applied knowledge search diversity, higher organizational proximity corresponds to a higher level of breakthrough innovation quality, supporting hypotheses H4b and H4.

### 4.3 Robustness checks

High-tech enterprises, characterized by knowledge intensity, technological advancement, and active innovation, are better equipped to reflect the impact of heterogeneous knowledge search on breakthrough technological innovation [26]. To verify the robustness of the research results, this study selected high-tech enterprises within the sample as a subsample for robustness checks. The specific results are shown in Tables 6 and 7. The results of the subsample hypotheses are consistent with those presented earlier, indicating that the empirical findings of this study are robust and reliable.

**Table 6:** Robustness check results for the quantity of breakthrough technological innovation.

Variables	Model 1	Model 2	Model 3	Model 4	Model 5
*FA*	0.0492^***^	0.0466^***^	0.0292^**^	0.0486^***^	0.0298^**^
*PS*	0.0001^**^	0.0001	0.0002^***^	0.0001^**^	0.0002^***^
*CS_YEAR*	0.1384^***^	0.0928^***^	0.0719^***^	0.0835^***^	0.0707^***^
*MAA_YEAR*	0.4458^***^	0.3218^***^	0.1911^***^	0.2806^***^	0.1836^***^
*BASIC_KSS*	–1.0096^***^	–1.4428^***^	–0.1035	–1.2864^***^	–0.1037
*APPLIED_KSS*	0.0853^***^	0.0948^***^	–0.0082	0.0850^***^	–0.0104
*BASIC_KSD*		5.1844^***^		5.4746^***^	
*BASIC_KSD^2^*		–3.9865^***^		–4.2220^***^	
*APPLIED_KSD*			3.5556^***^		3.5838^***^
*APPLIED_KSD^2^*			–1.9136^***^		–1.9425^***^
*OP*				0.3527^***^	0.6875^***^
*OP × BASIC_KSD*				–1.3831^***^	
*OP × BASIC_KSD^2^*				1.2422^**^	
*OP ×APPLIED_KSD*					–1.7682^***^
*OP × APPLIED_KSD^2^*					1.1015^***^
Constant	–2.1553^***^	–2.1776^***^	–2.7423^***^	–2.2181^***^	–2.7791^***^
Log likelihood	–3819.1393	–3689.0206	–3156.1833	–3655.5882	–3136.2758
Wald Chi-square	592.51	1009.65	1488.27	1067.59	1503.50
N	217	217	217	217	217

**Note:** ^*^p < 0.1, ^**^p < 0.05, ^***^p < 0.01.

**Table 7:** Robustness check results for the quality of breakthrough technological innovation.

Variables	Model 6	Model 7	Model 8	Model 9	Model 10
*FA*	0.0370^***^	0.0350^***^	0.0128^**^	0.0357^***^	0.0130^*^
*PS*	0.0001	–0.0001	–0.0001	–0.0001	–0.0001
*CS_YEAR*	0.1172^***^	0.0530^***^	0.0362^**^	0.0413^**^	0.0342^*^
*MAA_YEAR*	0.4409^***^	0.2757^***^	0.2035^***^	0.2383^***^	0.1851^***^
*BASIC_KSS*	–1.0868^***^	–1.5469^***^	0.0322	–1.3809^***^	0.0383
*APPLIED_KSS*	0.0925^***^	0.1008^***^	–0.0037	0.0909^***^	–0.0048
*BASIC_KSD*		6.1347^***^		6.2632^***^	
*BASIC_KSD^2^*		–4.5989^***^		–4.6452^***^	
*APPLIED_KSD*			5.1426^***^		5.3225^***^
*APPLIED_KSD^2^*			–3.3794^***^		–3.5007^***^
*OP*				0.3474^***^	0.8362^***^
*OP × BASIC_KSD*				–1.0585^***^	
*OP × BASIC_KSD^2^*				0.8781^*^	
*OP ×APPLIED_KSD*					–2.5683^***^
*OP × APPLIED_KSD^2^*					1.8377^***^
Constant	–2.4981^***^	–2.5450^***^	–3.2390^***^	–2.5745^***^	–3.2832^***^
Log likelihood	–4413.9298	–4262.704	–3702.8242	–4235.9614	–3681.1485
Wald Chi-square	583.21	1024.67	1335.36	1066.31	1351.10
N	217	217	217	217	217

**Note:** ^*^p < 0.1, ^**^p < 0.05, ^***^p < 0.01.

To ensure the robustness of the regression results and mitigate potential biases arising from differences in variable measurement and estimation methods, this study conducted several robustness checks: (1) re-measure dependent variable by adding highly cited utility model patents to the existing variable to better capture breakthrough technological innovation performance (as shown in Table 8); and (2) to reduce the impact of outliers, all micro-level continuous variables were winsorized at the 1% and 99% levels (as shown in Tables 9 and 10); (3) this study conducted empirical tests using a random-effects model (as shown in Tables 11 and 12). The results are highly consistent with those obtained from the previous analyses, further confirming the robustness of our findings.

**Table 8:** Robustness check results: re-measure dependent variable.

Variables	Model 1	Model 2	Model 3	Model 4	Model 5
*FA*	0.0121^***^	0.0143^***^	0.0126^***^	0.0146^***^	0.0132^***^
*PS*	0.0006^***^	0.0005^***^	0.0006^***^	0.0005^***^	0.0006^***^
*CS_YEAR*	0.3243^***^	0.2133^***^	0.1259^***^	0.2254^***^	0.1254^***^
*MAA_YEAR*	0.2583^***^	0.2012^***^	0.1422^***^	0.1914^***^	0.1367^***^
*BASIC_KSS*	0.1432	–0.5228^***^	–0.2952^***^	–0.5306^***^	–0.2970^***^
*APPLIED_KSS*	0.2598^***^	0.2555^***^	–0.0401^***^	0.2464^***^	–0.0462^***^
*BASIC_KSD*		2.4645^***^		2.9218^***^	
*BASIC_KSD^2^*		–1.2173^**^		–1.6793^***^	
*APPLIED_KSD*			4.4759^***^		4.1401^***^
*APPLIED_KSD^2^*			–2.8980^***^		–2.7170^*^
*OP*				0.0963^***^	0.3749^***^
*OP × BASIC_KSD*				–0.7520^***^	
*OP × BASIC_KSD^2^*				0.7267^**^	
*OP ×APPLIED_KSD*					–1.7842^***^
*OP × APPLIED_KSD^2^*					1.1514^***^
Constant	–2.0517^***^	–2.0605^***^	–2.2006^***^	–2.0555^***^	–2.1675^***^
Log likelihood	–4665.6150	–4597.4072	–4189.7465	–4583.8848	–4162.2303
Wald Chi-square	1543.47	1815.54	2228.11	1855.08	2257.21
N	280	280	280	280	280

**Note:** ^*^p < 0.1, ^**^p < 0.05, ^***^p < 0.01.

**Table 9:** Robustness check results: Winsorized treatment (the quantity of breakthrough technological innovation).

Variables	Model 1	Model 2	Model 3	Model 4	Model 5
*FA*	0.0119^***^	0.0140^***^	0.0113^***^	0.0144^***^	0.0125^**^
*PS*	0.0006^***^	0.0005^***^	0.0006^***^	0.0006^***^	0.0006^***^
*CS_YEAR*	0.3265^***^	0.2141^***^	0.1353^***^	0.2099^***^	0.1283^***^
*MAA_YEAR*	0.2473^***^	0.1923^***^	0.1312^**^	0.1867^***^	0.1214^**^
*BASIC_KSS*	0.1142	–0.5600^***^	–0.3332^***^	–0.5781^***^	–0.3389^***^
*APPLIED_KSS*	0.2810^***^	0.2770^***^	–0.0240^**^	0.2657^***^	–0.0316^***^
*BASIC_KSD*		2.5442^***^		3.0729^***^	
*BASIC_KSD^2^*		–1.3289^**^		–1.8600^***^	
*APPLIED_KSD*			3.9912^***^		3.5068^***^
*APPLIED_KSD^2^*			–2.3096^***^		–1.9764^***^
*OP*				0.1490^***^	0.4483^***^
*OP × BASIC_KSD*				–0.9388^***^	
*OP × BASIC_KSD^2^*				0.9105^***^	
*OP ×APPLIED_KSD*					–1.9503^***^
*OP × APPLIED_KSD^2^*					1.2078^***^
Constant	–2.2366^***^	–2.2492^***^	–2.4926^***^	–2.2449^***^	–2.4720^***^
Log likelihood	–4237.0025	–4171.6855	–3765.7943	–4153.6880	–3734.1369
Wald Chi-square	1533.69	1778.73	2028.43	18517.08	2045.87
N	280	280	280	280	280

**Note:** ^*^p < 0.1, ^**^p < 0.05, ^***^p < 0.01.

**Table 10:** Robustness check results: Winsorized treatment (the quality of breakthrough technological innovation).

Variables	Model 6	Model 7	Model 8	Model 9	Model 10
*FA*	0.0150^***^	0.0166^***^	0.0080^*^	0.0183^***^	0.0093^**^
*PS*	0.0003^*^	0.0001	0.0001	0.0001	0.0001
*CS_YEAR*	0.3558^***^	0.2600^***^	0.1496^***^	0.2555^***^	0.1556^***^
*MAA_YEAR*	0.2824^***^	0.1849^***^	0.1481^***^	0.1978^***^	0.1372^**^
*BASIC_KSS*	0.4406^***^	–0.2218^***^	0.0262	–0.2219^***^	0.0380
*APPLIED_KSS*	0.3112^***^	0.3087^***^	0.0261^**^	0.3001^***^	0.0197
*BASIC_KSD*		3.0941^***^		3.2891^***^	
*BASIC_KSD^2^*		–2.0773^***^		–2.0087^***^	
*APPLIED_KSD*			5.1993^***^		4.8180^***^
*APPLIED_KSD^2^*			–3.4255^***^		–3.1125^***^
*OP*				0.1034^***^	0.4583^***^
*OP × BASIC_KSD*				–0.4694	
*OP × BASIC_KSD^2^*				0.1781	
*OP ×APPLIED_KSD*					–2.3857^***^
*OP × APPLIED_KSD^2^*					1.6099^***^
Constant	–2.9189^***^	–2.9415^***^	–3.3394^***^	–2.9740^***^	–3.3277^***^
Log likelihood	–4899.8237	–4835.4883	–4453.0112	–4823.5764	–4428.3256
Wald Chi-square	1701.72	1887.32	1802.52	1910.87	1796.26
N	280	280	280	280	280

**Note:** ^*^p < 0.1, ^**^p < 0.05, ^***^p < 0.01.

**Table 11:** Robustness check results: Random-effects model (the quantity of breakthrough technological innovation).

Variables	Model 1	Model 2	Model 3	Model 4	Model 5
*FA*	0.0171^***^	0.0174^***^	0.0091^***^	0.0182^***^	0.0100^***^
*PS*	0.0002^***^	0.0001^***^	0.0002^***^	0.0002^***^	0.0002^***^
*CS_YEAR*	0.1907^***^	0.0998^***^	0.0789^***^	0.0936^***^	0.0770^***^
*MAA_YEAR*	0.4254^***^	0.3017^***^	0.2063^***^	0.2621^***^	0.2000^***^
*BASIC_KSS*	–1.0744^***^	–1.5955^***^	–0.1233^*^	–1.4348^***^	–0.1248^*^
*APPLIED_KSS*	0.0912^***^	0.1030^***^	–0.0227^**^	0.0925^***^	–0.0258^***^
*BASIC_KSD*		5.9046^***^		6.1511^***^	
*BASIC_KSD^2^*		–4.2841^***^		–4.5383^***^	
*APPLIED_KSD*			4.3000^***^		3.8159^***^
*APPLIED_KSD^2^*			–2.4074		–2.1398
*OP*				0.3070^***^	0.4121^***^
*OP × BASIC_KSD*				–1.4189^***^	
*OP × BASIC_KSD^2^*				1.3266^***^	
*OP ×APPLIED_KSD*					–1.7997^***^
*OP × APPLIED_KSD^2^*					1.1246^***^
Constant	–1.6853^***^	–2.0605^***^	–2.5158^***^	–1.8253^***^	–2.5050^***^
Log likelihood	–6116.9099	–5885.5467	–5084.0734	–5823.9317	–5052.5066
Wald Chi-square	859.56	1576.88	2372.91	1738.99	2376.06
N	280	280	280	280	280

**Note:** ^*^p < 0.1, ^**^p < 0.05, ^***^p < 0.01.

**Table 12:** Robustness check results: Random-effects model (the quality of breakthrough technological innovation).

Variables	Model 6	Model 7	Model 8	Model 9	Model 10
*FA*	0.0183^***^	0.0181^***^	0.0102^***^	0.0190^***^	0.0110^***^
*PS*	0.0001^*^	–0.0001	–0.0001	–0.0001	–0.0001
*CS_YEAR*	0.1736^***^	0.0766^***^	0.0466^***^	0.0623^***^	0.0446^***^
*MAA_YEAR*	0.4685^***^	0.2744^***^	0.2186^***^	0.2383^***^	0.2072^***^
*BASIC_KSS*	–1.1130^***^	–1.6587^***^	0.0652	–1.4975^***^	0.0713
*APPLIED_KSS*	0.0969^***^	0.1074^***^	–0.0081	0.0971^***^	–0.0092
*BASIC_KSD*		7.0423^***^		6.9768^***^	
*BASIC_KSD^2^*		–5.2568^***^		–4.9972^***^	
*APPLIED_KSD*			4.9563^***^		4.5263^***^
*APPLIED_KSD^2^*			–2.8310^**^		–2.6004
*OP*				0.3000^***^	0.4134^***^
*OP × BASIC_KSD*				–1.1240^***^	
*OP × BASIC_KSD^2^*				0.8395^*^	
*OP ×APPLIED_KSD*					–2.0765^***^
*OP × APPLIED_KSD^2^*					1.4272^***^
Constant	–2.3187^***^	–2.4708^***^	–3.3904^***^	–2.4932^***^	–3.3833^***^
Log likelihood	–7094.2532	–6838.0428	–6024.8789	–6786.4518	–6000.4494
Wald Chi-square	905.93	1705.62	2049.80	1813.33	2037.70
N	280	280	280	280	280

**Note:** ^*^p < 0.1, ^**^p < 0.05, ^***^p < 0.01.

## 5 Conclusion and Implications

### 5.1 Research findings

This study utilizes patent data and panel data from 280 Chinese new energy vehicle (NEV) manufacturing firms between 2010 and 2019, employing a fixed-effects negative binomial regression model to test hypotheses and examine the relationship between various knowledge diversity search behaviors and firms’ breakthrough technological innovation. The primary findings are as follows:

Firstly, knowledge search diversity exhibits an inverted U-shaped relationship with breakthrough technological innovation. As firms increase the diversity of their knowledge search, their capacity for breakthrough innovation initially improves. However, the marginal returns from such diversity decline over time, and beyond a certain threshold, the effect turns negative. For instance, when the diversity of basic knowledge search is below 0.681 (*x* = −*b*/2*a*), increasing diversity contributes to greater heterogeneity within the firm’s knowledge base, significantly enhances the potential for knowledge recombination, and promotes the development of breakthrough innovations. Once this threshold is exceeded, however, the firm may face escalating search costs and mounting cognitive pressure to absorb redundant or overlapping knowledge, which can impede the effective translation of innovative outcomes. A similar trend is observed in the relationship between applied knowledge search diversity and breakthrough innovation. These results underscore the role of knowledge search diversity as a critical foundation for effective knowledge recombination. In practice, firms can adopt diverse search strategies—such as engaging in industry-academia collaborations or establishing strategic alliances—to broaden their knowledge base and enhance both basic and applied innovation capabilities. Nevertheless, due to the diminishing marginal returns of knowledge search diversity, firms with limited resources and absorptive capacity must strike a balance: excessive investment in the early-stage search process may drain organizational attention and resources, ultimately compromising momentum for later-stage innovation. This conclusion is consistent with Rajalo *et al*. (2021), who found that moderate knowledge search diversity enhances innovation performance, while overly extensive open search, especially under resource constraints, may adversely affect innovation outcomes.

Secondly, compared with applied knowledge search diversity, basic knowledge search diversity demonstrates a steeper inverted U-shaped relationship with breakthrough technological innovation. This suggests that breakthrough innovation often requires fundamental changes or radical reinterpretations of the principles underlying existing technologies or products. Basic knowledge search provides firms with the theoretical foundations and scientific rationale necessary for such transformations, thereby offering greater inspiration and support for initiating breakthrough innovation. Moreover, while firms are generally more adept at applied technologies, they often lack strong capabilities in basic research. In this context, a diverse search for basic knowledge can effectively fill gaps in firms’ internal knowledge bases, leverage knowledge complementarity, and enhance their potential for breakthrough innovation. These findings support the argument put forward by Della *et al*. (2015), who contend that firms engaging with basic science are more likely to generate breakthrough inventions.

Thirdly, organizational proximity positively moderates the inverted U-shaped relationship between knowledge search diversity and breakthrough technological innovation. As shown in Figs 2, 3, 4, 5, in the ascending segment of the curve—before the inflection point—higher levels of organizational proximity are associated with greater breakthrough innovation performance under the same level of knowledge search diversity. This indicates that, during innovation processes facilitated by collaboration, firms with higher organizational proximity are more capable of engaging in breakthrough technological activities. High organizational proximity among partners fosters shared goals and a common belief system, which creates a favorable environment for effective communication and mutual learning. Trust among innovation actors facilitates the smooth flow of knowledge and enhances opportunities for diverse knowledge recombination. Furthermore, strong organizational proximity improves the efficiency of inter-organizational knowledge exchange and collaboration, enabling more effective knowledge integration, which significantly contributes to the realization of breakthrough technological innovations. For instance, Huawei’s extensive R&D system exemplifies this mechanism: departments across the organization align on common research objectives and regularly engage in cross-departmental exchanges, promoting the integration and sharing of knowledge. This structure not only accelerates the development of breakthrough technologies but also helps the firm avoid critical technological bottlenecks.

### 5.2 Theoretical contributions

The main theoretical contributions of this study are as follows: Firstly, from the perspective of the relationship between knowledge and industry, this study analyzes the knowledge search strategies adopted by firms in the new energy vehicle (NEV) industry to seize development opportunities and achieve breakthrough technological innovations. Unlike previous research on knowledge search and breakthrough innovation, which predominantly focuses on overcoming organizational, technological, or geographical boundaries [1,2,20], this study enriches and extends knowledge search theory by classifying the diversity of knowledge searches based on the perspective of knowledge system reconstruction. This theoretical contribution enhances the understanding of knowledge search diversity and offers valuable insights for firms engaged in breakthrough technological innovation practices.

Secondly, in contrast to the case-based studies typically conducted in the domain of complex technological products [30], this study uses a fixed-effects negative binomial regression model to empirically examine the NEV industry. By doing so, it clarifies the mechanisms through which knowledge search diversity influences breakthrough technological innovation. The results offer broader generalizability, objectivity, and applicability, providing solid theoretical support for firms seeking to engage in breakthrough technological innovation activities.

Thirdly, integrating complex product systems theory and transaction cost theory, this study highlights the role of organizational proximity in shaping the relationship between knowledge search diversity and breakthrough technological innovation. From a knowledge management perspective, it offers new avenues and practical guidelines for firms in complex technological sectors to strategically select proximate organizations for knowledge search, thereby accelerating the achievement of breakthrough technological innovations.

### 5.3 Practical recommendations for management

Building on the above conclusions, this study provides the following managerial recommendations for firms seeking to engage in knowledge search diversity and drive breakthrough technological innovation: Firstly, the success of technological innovation often requires composite talents, and firms should focus on developing diversified skills among their employees to meet the challenges of technological convergence. Firms should encourage R&D personnel to independently develop solutions and processes tailored to specific challenges, supporting the acquisition of diverse and heterogeneous knowledge. This can be achieved by providing access to various platform-based information resources, regularly participating in industry seminars, and engaging in industry-academia-research collaborations to obtain cutting-edge scientific knowledge and technological support. These efforts can help prevent firms from falling into capability traps and foster the generation and conversion of creative, disruptive ideas.

Secondly, with the rapid iteration of technology, firms need to develop flexible knowledge search plans and adjust their search direction in time to adapt to market changes. While basic research is the cornerstone of technological innovation and plays a crucial role in driving breakthrough innovations, its translation into applied technologies typically involves longer cycles and higher risks. Therefore, firms should actively invest in basic knowledge search diversity when financial resources permit them to establish long-term technological advantages. During the early stages of development, however, firms should prioritize the search for application knowledge search diversity, which helps to reduce uncertainties and risks associated with the translation process, ensuring steady growth while simultaneously pursuing breakthrough innovations.

Thirdly, strategic partnerships should be established to fully access and exploit the fundamental and applied knowledge of investment holding companies. The strong alignment of interests in these relationships fosters trust, ensuring that knowledge search diversity is deeply integrated while minimizing the risk of knowledge leakage. Furthermore, organizational proximity enhances collaborative synergies between firms, accelerating the realization of novel ideas. Specifically, when seeking industry knowledge, firms should prioritize high organizational proximity, as this ensures the full acquisition and comprehension of cutting-edge application knowledge, which in turn supports the rapid conversion of breakthrough innovations.

In addition, to enhance the practical relevance of our findings, we have proposed the following recommendations for firms at different stages of development, scales, and industries: Mature enterprises should focus on managing the diversity of their R&D portfolios, guiding strategic cooperation and external knowledge absorption, as well as facilitating the diversity and flow of knowledge through the establishment of cross-functional collaboration platforms. Medium-sized enterprises, which may be more limited in resources, should flexibly adjust their knowledge search strategies in R&D activities and focus on creating an innovation-orientated corporate culture, which can be enhanced through internal cross-functional knowledge sharing and cooperation with external partners. And since start-ups usually have limited resources, start-ups focus on core technologies and market positioning, avoiding excessive upfront investment with agile innovation and rapid experimentation, while actively introducing external knowledge with cooperation.

In the health technology sector, R&D teams can leverage knowledge search diversity to explore novel medical device technologies. For instance, a company specializing in smart medical devices may collaborate with experts across disciplines—such as materials scientists, data analysts, and clinicians—to broaden its technological horizons. This cross-disciplinary knowledge search can facilitate the discovery of new material applications or data analytics methods, ultimately leading to the development of more precise and efficient medical devices. For R&D managers in digital health, our findings suggest proactively establishing cross-institutional partnerships (e.g., hospital-university joint laboratories) to leverage ownership proximity for accelerating knowledge transfer related to FDA compliance.

In the advanced manufacturing industry, strategic leaders can utilize knowledge search diversity to optimize production processes. For example, an automotive manufacturer may collaborate with software firms to incorporate advanced data analytics technologies into its production lines. Such partnerships can not only enhance production efficiency but also reduce waste and defect rates. Strategic leaders in the electric vehicle battery manufacturing industry can minimize the tacit knowledge gap in the design of thermal management systems by establishing industrial alliances.

To further assist industry leaders in applying these insights across contexts, we propose several reflective questions to encourage alignment between our research findings and firm-specific realities: (1) How might your organization map existing structural proximities (e.g., equity stakes, board interlocks) to identify untapped knowledge flow opportunities? (2) What governance mechanisms (e.g., rotation programs, alliance KPIs) could amplify proximity effects in your sector?”

### 5.4 Research limitations and future directions

While this study focuses on knowledge search diversity and provides new theoretical insights for enhancing breakthrough technological innovation, it has several limitations. First, the findings are more applicable to industries characterized by high technological complexity and systemic integration. Future research could incorporate environmental variables such as technological turbulence to extend the study to other industries. Second, the sample predominantly consists of private NEV firms, resulting in data limitations regarding firm characteristics. Future studies could employ surveys or other methods to enhance the diversity and accuracy of data sources. Finally, the process of knowledge creation is influenced by more than just knowledge search; factors such as top management team characteristics also play a significant role in R&D decision-making. Future research could further explore related variables to refine the pathways and mechanisms through which firms achieve breakthrough technological innovation.
